# Ethnobotanical study of traditional medicinal plants used to treat human ailments in West Shewa community, Oromia, Ethiopia

**DOI:** 10.3389/fphar.2024.1369480

**Published:** 2024-07-19

**Authors:** Tamirat Bekele Beressa, Diriba Alemayehu Gadisa, Siraj Mammo, Gurmu Tesfaye Umeta, Lemma Bose Meskele, Biruk Mosisa Gudeta, Getu Melesie Taye

**Affiliations:** ^1^ Department of Pharmacy, College of Medicine and Health Sciences, Ambo University, Ambo, Ethiopia; ^2^ Department of Biology, College of Natural and Computational Science, Ambo University, Ambo, Ethiopia

**Keywords:** ethnobotanical study, traditional medicine, medicinal plants, West shewa, Ethiopia

## Abstract

**Introduction:** Plants have formed the basis of traditional medicine (TM) systems, which have been used for thousands of years. According to reports, one-quarter of the commonly used medicines contain compounds isolated from plants. This study aims to identify and document the plants for ethno-pharmacological use by the indigenous communities of West Shoa Zone, Oromia region, Ethiopia.

**Methods:** The cross-sectional study was conducted from November 2020 to November 2021 in West Shewa Zone, Oromia Region, Ethiopia. The ethnobotanical data was collected from Ejere District, Ada Berga District, Dandi District, Ambo District, Ambo Town, Toke Kutaye District, and Bako Tibe District. A descriptive statistical method (percentage and/or frequency) was employed to summarize ethnobotanical data. Moreover, the informant consensus factor was computed. Microsoft Excel spreadsheet software (Microsoft Corporation, 2016) and SPSS (version 25) were used to organize and analyze the data.

**Result:** In the study area, a total of 51 families of medicinal plants with 108 Species were identified. Fabaceae 8 species, Asteraceae, Solanaceae and Lamiaceae each with 6 species and Cucurubitacieae 5 species were the frequently reported medicinal plants. The leaf (57.2%) was the most widely used medicinal plant parts, and oral administration (56.5%) was the most cited route of administration. In the present study, most of the medicinal plants were used fresh, which was (75%) and the most common disease the healers treated was gastrointestinal disease, followed by skin disease and febrile illness. The major threat to medicinal plants in the study area was agricultural expansion, which was reported by 30.6% of the respondents. The study area was rich in medicinal plants, Fabaceae which commonly used family.

**Conclusion:** Most of the medication prepared by the traditional healers was taken orally and derived from the leaf part of the medicinal plant. Since this research is a preliminary study which will be used as a base for further study. The efficacy and safety of the medicinal plant claim should be studied in the future.

## Introduction

The use of natural products as medicinal agents dates back to prehistory. According to a World Health Organization (WHO) report, 60%–79% of the population in developing countries depends on traditional medicine from plants for health requirements (WHO, 2010). The use of indigenous traditional medicine in Ethiopia is also estimated between 60% and 79% (World Health Organization, 2019). The Ethiopian people have been using medicinal plants to treat different diseases for many centuries. Religious and secular pharmacopeia has been compiled since the 15th century. Medicinal plants are an integral part of the variety of cultures in Ethiopia, which has resulted in medical system pluralism (Pankhurst, 1965).

According to reports, one-quarter of the commonly used medicines contain compounds isolated from plants (Rates, 2001). The search for new drugs is a priority of WHO, drug companies, and research institutes due to the emergence and spread of drug-resistant pathogens that have acquired new resistance mechanisms, leading to antimicrobial resistance, which continues to threaten our ability to treat common infections (WHO Pathogens Priority List Working Group et al., 2018). Especially alarming is the rapid global spread of multi- and pan-resistant bacteria (also known as “superbugs”) that cause infections that are not treatable with existing antimicrobial drugs. Emerging and reemerging infectious diseases continue to impose a constant threat on the human population (Howard and Fletcher, 2012).

Even though the national policy of Ethiopia indicates identifying and encouraging the utilization of beneficial TM components (EFDA, 2020), little has been done to enhance and develop the beneficial aspects of TM, including relevant research to explore possibilities for its gradual integration into modern medicine (Kassaye et al., 2006). Indigenous knowledge is usually kept secret, only to be passed orally to the healer’s older son at their oldest age (Battiste and Youngblood, 2000). The expansion of modern education has also impacted the transfer culture, and knowledge on medicinal plants is being lost at a faster rate (IBCR, 2001). Additionally, the loss of medicinal plants is due to population pressure, agricultural expansion, and deforestation (Abebe et al., 2001; Berhan and Dessie, 2002).

Due to changing lifestyles, extreme secrecy of traditional healers, and negligence of youngsters, the practice and dependence of ethnic societies on folk medicines are in rapid decline globally (Kumar et al., 2012). Therefore, documentation of the traditional uses of medicinal plants needs immediate attention (Bussmann and Sharon, 2006; Jeyaprakash et al., 2017). Furthermore, documentation of indigenous and traditional knowledge is very important for future critical studies leading to sustainable utilization of natural resources and facing the challenges of biopiracy and patenting indigenous and traditional knowledge by others. Therefore, it is urgent to explore and document this unique indigenous and traditional knowledge of the tribal community before it diminishes with the knowledgeable persons. This study aimed to identify and document the plants for ethno-pharmacological use by the indigenous communities of the West Shoa, located in the Oromia regional state, Ethiopia.

## Methods

### Study area description

#### Geographical location

**FIGURE 1:**
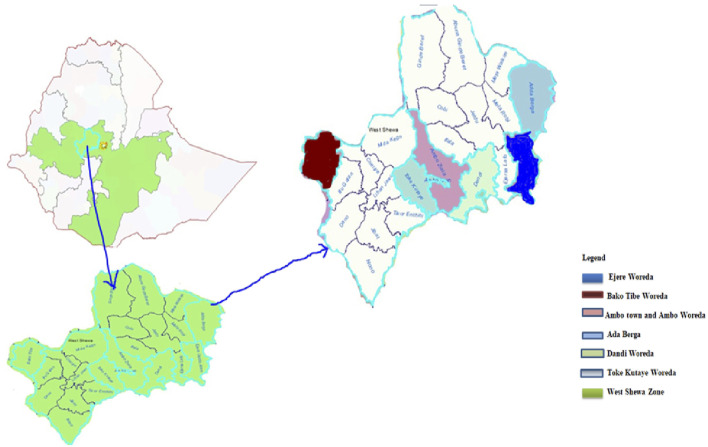
Map of the study area.

The study was conducted in the West Shoa Zone which is found in Oromia Region, Ethiopia. West Shewa is bordered on the South by the Southwest Shewa Zone, on the Southwest by Jimma Zone, on the West by East Wolega, on the Northwest by Horo Gudru Welega, on the North by the Amhara Region, on the Northeast by North Shewa, and from the East by Sheggar City (Figure 1). The district is located between 8°49′26″to 8°55′22″N and 37°49′50″ to 38°8′08″E (Ogato et al., 2020). Based on the agro-climatic classification of Ethiopia, it lies within the three agro-climatic zones, Highland 27%, mid-altitude 56% and 17% lowlands. The zone has a bimodal rainfall pattern; summer is the main rainy season with its peak in July (June to August), and the short rainy season from February to April. Rainfall varies from 813.2 mm to 1669.1 mm. The maximum temperature ranges from 24°C to 29°C while the minimum temperature ranges from 11°C to 13°C (Abate, 2008).

### Study design and period

A cross-sectional survey was conducted from November 2020 to November 2021.

### Site and informant selection procedure

Seven Woredas and one town were purposively selected. Accordingly, Ambo town, Ejere, Ambo Woreda, Ada Berga, Bako Tibe, Toke Kutaye, and Dandi Woredas were selected (Figure 1). From each woredas traditional healers or knowledgeable persons were selected based on the information gathered from each selected kebele administration office, health office, agricultural office, and other people in the study area.

### Selection of informants

The selection of key informants was conducted by snowball method with the help of local authorities, elders, and knowledgeable persons. All registered traditional healers and unregistered but famous elders and knowledgeable persons within each woreda were included. Prior to the administration of the questionnaire, conversations with the informants were done with the assistance of local leaders of the selected study area to elaborate the objectives of the study and to build on trust with the common goal of documenting and preserving knowledge on medicinal plants.

### Ethno-medicinal data collection and tools

Fourteen data collectors participated after 5 days of training. Semi-structured questionnaires, Focus group discussions, and field observation were used for data collection. The tool for data collection was developed by reviewing previous research (Yirga and Zeraburk, 2011; Chekole, 2017), and modified to fit the local population. The questionnaire was designed in English and translated by professionals to the Afan Oromo. Questionnaire content validation was done by pretest. Content Information regarding local names of medicinal plants, methods of preparation, part(s) used, dosage used and route of administration of medicinal plants, types of disease, and duration of administrations were recorded.

### Field observation

Field observation was performed with the help of local guides and interviewed informants in the study area. Based on the information provided by informants, specimens were collected, numbered, pressed, and dried for identification.

### Focus group discussion

Focus group discussions were conducted for the selected district with the community members who have used medicinal plants from well-known traditional healers in the community.

### Plant identification

Voucher specimens were collected for each plant species. The specimens were dried, pressed, and identified in the National Herbarium (ETH), Addis Ababa University. Finally, the identified voucher specimens were deposited at the National Herbarium (AAU).

### Data quality management

The quality of data was maintained by providing training for data collectors, regarding the purpose of the study and data handling. The collected data was checked for completeness and consistency before analysis of data.

### Data analysis

A descriptive statistical method (percentage and/or frequency) was employed to summarize ethnobotanical data. Moreover, the informant consensus factor was computed. Microsoft Excel spreadsheet software (Microsoft Corporation, 2016) and SPSS (version 22) were used to organize and analyze the data.

Informant consensus factor (ICF) was calculated for categories of ailments to identify the agreements of the informants on the reported cures using the formula used by Gazzaneo, de Lucena (Gazzaneo et al., 2005) and Teklehaymanot and Giday (Teklehaymanot and Giday, 2007). ICF was calculated as follows:

$$\text{IFC}=\frac{Nuc-Ns}{Nuc-1}$$

Where Nuc is the number of use citations in each illness category and Ns = is the number of species used by all informants for this illness category. The ICF values range from 0 to 1, with high values (i.e., close to 1) indicating that relatively few plants are used by a large proportion of informants, while low values (˂ 0.5) indicate that informants do not agree on the plant species to be used to treat a category of ailments.

### Ethical consideration

The ethical clearance was obtained by the Ethical Review Committee of the College of Medicine and Health Sciences of Ambo University. Then the letter written from Ambo University was given to the West Shewa Zone health office. The West Shewa zone health office wrote a supportive letter to each Woreda Health office. In addition, oral informed consent was obtained and those who voluntarily participated was continued the interview. The confidentiality was assured by excluding their names and the right not to participate or to discontinue the interview wherever they wanted in the study was respected.

## Results

### Socio-demographic characteristics

From six woredas and one administrative town a total of 126 participants responded to the interview. Out of the participants 79.4% were male and the majority of the participants were Orthodox and Oromo. The more than two-third of the participants (78%) were greater than 50 years. More than 80% of the participants had the experience of giving traditional medicine greater than 10 years. More than eighty percent of the participant got the knowledge about traditional medicine from the family members (Table 1).

**TABLE 1:** Sociodemocratic characteristics of respondents of Southwest Shewa zone.

Variables	Categories	Number (N = 126)	Percent (%)
Sex	Male	100	79.4
	Female	26	20.6
Resident of participant	Baco Woreda	20	15.8
	Ejere Woreda	20	15.8
	Toke Kutaye Woreda	20	15.8
	Ambo town	12	9.5
	Ada Berga Woreda	16	12.7
	Ambo Woreda	20	15.9
	Dandi Woreda	18	14.3
Religion	Protestant	34	26.9
	Ortodox	70	55.5
	Muslim	18	14.3
	Waqefata	4	3.2
Ethnicity	Oromo	118	93.7
	Amhara	8	6.3
Educational Status	Could not read and write	60	47.6
	Can read and write	6	4.8
	Primary School (1–8)	51	40.5
	Secondary school (9–12)	9	7.1
Job	Farmer	96	76.2
	Gov. employer	22	17.5
	Private work	8	6.3
Source of Knowledge	Family	112	88.9
	Friend	10	7.9
	Traditional healer	4	3.2
Pattern of work	Pert-time	118	93.7
	Full time	8	6.3
Age	<50	48	38.1
	≥50	78	61.9
Number of patient/week	<10	114	90.5
	>10	12	9.5
Experience in years	<10	40	31.7
	>10	86	68.3

### Medicinal plant family and species

In the study area a total of 51 families of medicinal plants with 108 species were identified. Fabaceae (8 species), Asteraceae (6 species), Solanaceae (6 species), Lamiaceae (6 species), and Cucurubitacieae (5 species) were the most frequently reported medicinal plant in the study area (Supplementary Table S1).

### Plant parts used

**FIGURE 2:**
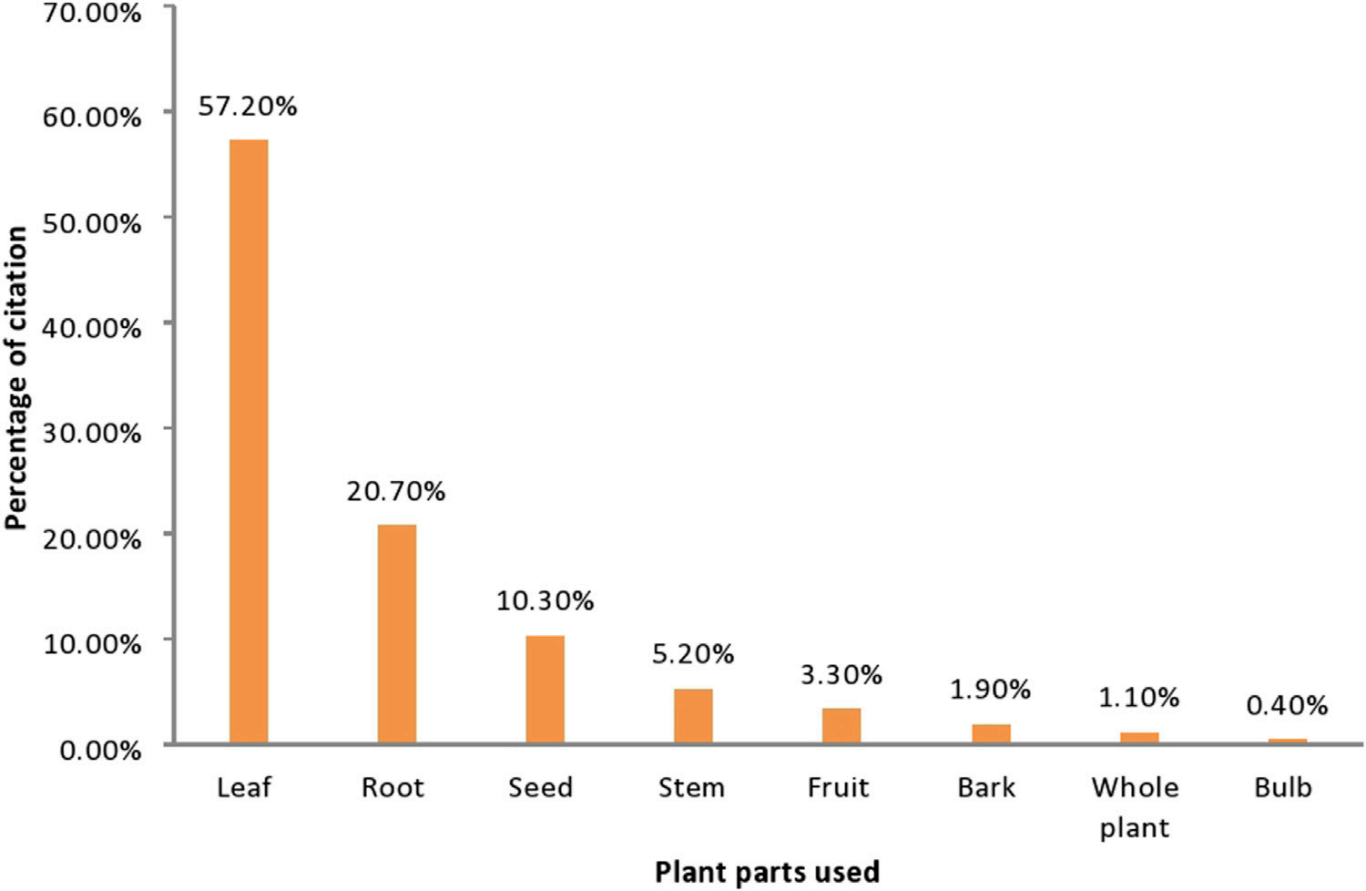
Part the medicinal plants used for medicine by traditional healers in West Shewa, Oromia, Ethiopia.

In the present the leaf (57.2%) was the most widely used medicinal plant parts followed by the root which was 20.7% (Figure 2).

### Route of administration of the remedies prepared from medicinal plant and condition of medicinal plant

The oral administration was the most (56.5%) cited route of administration followed by the topical (34.3%), inhalation (6.3%) and nasal (2.9%) route of administration. On the other hand most of the medicinal plants were used as fresh which was (75%) and 22% of the medicinal plants were used after drying the rest (3%) can be utilized both in the form of dry and fresh.

### Method of preparations

**FIGURE 3:**
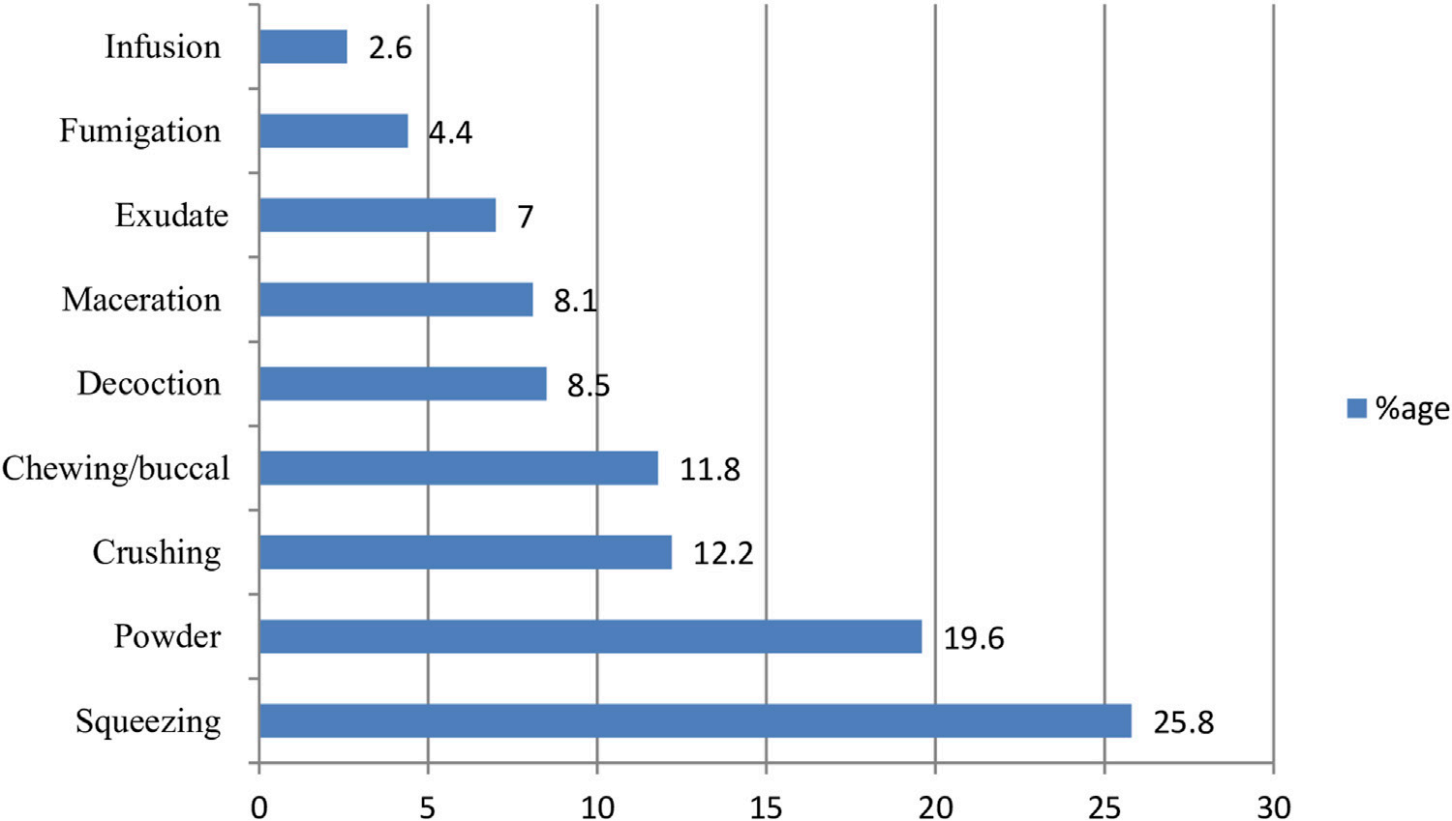
The method medicine preparations used by the traditional healers in West Shewa, Oromia, Ethiopia.

In the present study the majority of the medicine were prepared by squeezing (25.8%) followed by powder (19.6%) (Figure 3).

### Additive during medicine preparation

Out of 126 participants 120 (95.2%) prepare the medicine in combination with other medicinal plant or non-medicine ingredients such as water, honey, milk, and food. Out of the additives used 102 (80.9%) of the participants were used medicinal ingredient and 18 (14.3%) were ingredient with non-medicinal value but used as excipient.

### Saying during plant collection

During plant collection majority 84 (66.67%) of the participants did not talk and 42 (33.33%) pray to their God before collection.

### Storage and packaging material for medicine

The majority of the participants used plastic bag (81 reports) followed by bottle (60 reports), fasten with clothes (27 reports), putting in cabinet (6 reports) and few of them use sack (3 reports) for storage and packaging of medicine after preparation.

### Disease category and informant consensus factor

The most common disease the healers treat were gastrointestinal disease followed by skin disease and febrile illness (Table 2).

**TABLE 2:** Disease category and ICF of Medicinal plants used by traditional healers in West Shewa, Oromia, Ethiopia.

Disease category	No species	No of reports	ICF
Tumour	2	5	0.75
Rabies	6	19	0.7
Febrile illness	12	31	0.63
STI (Gonorrhoea, hepatitis, herpes simplex, Syphilis	5	11	0.6
Skin disease (Allergic reaction, wound healing, skin disorder, tinea versicolar, ring worm, scabies, Eczema, leprosy, Broken bone, dislocation, wart, itching, desert wound, burn)	18	38	0.54
Haemorrhoid	6	11	0.5
Tonsillitis	10	18	0.47
Abdominal cramp/distension/stomacache/tape worm/diarrhoea/dyspepsia/indigestion, ascariasis, constipation	29	50	0.43
Toothache	9	15	0.43
Common cold/Cough, Asthma, respiratory disease	7	11	0.4
Leshmaniasis, measles, herpes simplex	11	17	0.4
Anthrax	11	14	0.23
Others (Ear pain, Eye pain, emergency, evil eye, evil spirit, impotency)	10	12	0.18
Cardiovascular (Diabetes mellitus, edema, hypertension, kidney disease, Swelling, stop bleeding, urinary retention)	14	14	0
Poisoning (snake and Spider)	4	4	0

### Duration of the medication storage after preparation and action taken for treatment failure

The mean of the expired date of the medication after preparation was 6.67 months with a range of 1 month–24 years. The majority of the participants (118, 89.6%) reported that their medicine is effective and would treat the patients. The rest of the participants 6.8% reported that would advise the patient to visit modern health facilities if the treatment failed and other said they will increase a dose. The figures cited here may not accurately represent reality, as it is based on self-reported data, and their validity could not be verified due to its nature.

### Threats to medicinal plants

**FIGURE 4:**
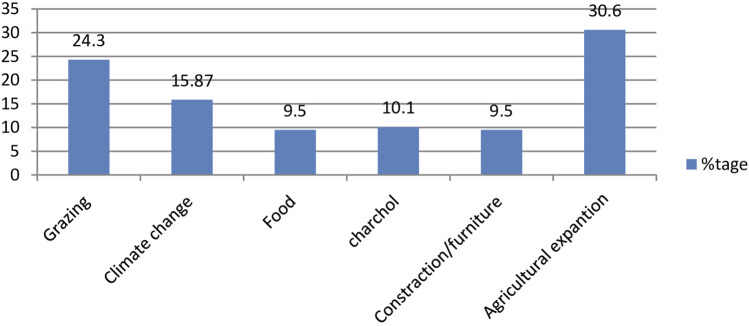
Treats to medicinal plant by traditional healers in West Shewa, Oromia, Ethiopia.

The major threat to medicinal plant in the study area was agricultural expansion which was reported by 30.6% of the respondents (Figure 4).

## Discussions

More than two-thirds of the participants were male, and the majority of the participants were greater than 50 years old. The study conducted in Jimma Zone, Ethiopia, showed males were the majority who participated as traditional healers (Yineger and Yewhalaw, 2007). This may be due to the traditional knowledge in the community being passed from male parents to the firstborn (Bishaw, 1990). Moreover, the elders were the community members who had knowledge of medicinal plants, which was also in line with the study conducted by Chekole (Chekole, 2017). It was also reported in the study conducted in southern Brazil by Meretika et al. (Meretika et al., 2010) that the elders have greater knowledge of medicinal plants. In the present study, the majority of the participants obtained their knowledge of traditional medicine from family members. This was in line with the study conducted in the central zone of Tigray, which revealed that indigenous knowledge was only transferred to the selected family members (Gidey, 2010).

The study area was rich in medicinal plants (108 medicinal plants species) were identified and this showed still large number of the community member depends on the traditional medicinal plants for their healthcare needs. In the study area, Fabaceae was the most frequently cited medicinal plant family, followed by Asteraceae, Solanaceae, Lamiaceae, and Cucurbitaceae. This was in line with the study conducted in different parts of Ethiopia (Bekalo et al., 2009; Megersa et al., 2013; Alebie and Mehamed, 2016; Kebebew, 2016) and in other countries in Africa (Kambizi and Afolayan, 2001).

The leaves were the most frequently cited plant parts used, followed by the root. This agrees with other findings conducted in Ethiopia (Awas and Demissew, 2009; Eshete et al., 2016; Shimels et al., 2017; Weckmüller et al., 2019), as leaves were frequently plant parts used in the traditional remedy preparations. Because of the numerous potential dangers that surround them, plants have evolved defense mechanisms to fend off herbivores, pathogens (such as bacteria, viruses, fungus, nematodes, mites, and insects), and diverse abiotic stresses (Teklay et al., 2013). As a result, plants generate a variety of organic substances known as secondary metabolites, which are inherently unrelated to the growth and development of the plants (Piasecka et al., 2015). It may be argued that since the leaves are more exposed to the enemy, these compounds serve as a form of defense (Bartwal et al., 2013). Therefore, the use of leaves as a primary component of a plant in a medicine preparation could be regarded as a sign of scientific validity. It is also important to note that the discovery of several aliphatic medicines was historically attributed to the zoopharmacognostic approach, which involves the monitoring of animal self-medication behavior. This further supports the preference for using leaves as a source of TMs (Shurkin, 2014; Tuasha et al., 2018). The preference for leaves over other plant components may be attributed to the ease with which they can be prepared in comparison to medicine formulations from roots, stem barks, whole plants, and seeds.

The oral route of administration was the most commonly used way of administrating of medicinal plants in the study area, followed by topical administration. The finding regarding administration routes corresponds to the finding made by Mesfin et al. (Mesfin et al., 2013) and Tolossa et al. (Tolossa, 2007). The oral route of administration was significantly higher than other methods in the study community, most likely because the most prevalent disease in the area is associated with internal disorders such as stomachaches, intestinal parasites, tonsillitis, and others, for which oral administration was more efficient. Furthermore, oral administration may be connected with a significant contribution to a quick physiological response to the causative agents, enhancing the therapeutic potential of traditional medicinal herbs.

The fresh 74.9% was the most commonly used condition of medicinal plant. This was in line with the study conducted in different parts of Ethiopia (Gidey et al., 2011; Mengesha, 2016; Hordofa and DGNRA, 2017; Eshete and Molla, 2021).

Squeezing (25.8%) was the most commonly used method of preparation, followed by powder which accounts 19.6%. The finding was in line with the study conducted by (Getnet et al., 2016). However, unlike the present study, Mengesha et al. (Mengesha, 2016) and Fenetahun et al. (Fenetahun et al., 2017) reported crushing as the most commonly reported method of medicinal plant preparation.

The majority of the participants, 95.2%, prepare the medicine in combination with other medicinal plants or non-medicine ingredients. Out of the additives, 80.9% were added for its medicinal purposes. Non medicinal ingredients like water, honey, milk, and food were utilized in formulating the medicine. The findings of the present study was in line with the study conducted in Jimma Zone which reported most of the additives reported were medicinal plant which increase the effectiveness (Abera, 2003; Yineger and Yewhalaw, 2007).

The majority of the dried medicinal plants were stored in a plastic bag, followed by a bottle. This finding was in line with the study conducted by Chekole (Chekole, 2017) revealed that plastic bags and cloths were mainly used for the storage of dried medicinal plants. The other studies conducted in different districts in Ethiopia also revealed that plastic bags were used as a preservation method (Abera, 2003; Tesfaye and Erena, 2020; Kindie et al., 2021).

The most common diseases treated by medicinal plants reported by the participants were gastrointestinal diseases followed by skin diseases. This was in line with the study conducted by Teklehaymanot and Giday (Teklehaymanot and Giday, 2007) that showed the largest number of remedies were used to treat gastrointestinal disorder followed by external injuries (skin disorder). The other study conducted in Debrelibanos district also revealed that the highest number of plant species were used to treat gastrointestinal disorders like abdominal pain, intestinal parasites, and diarrhea (Seyoum and Zerihun, 2014).

The informant consensus factor indicates the agreements of the informants on the reported cures for the group of diseases (Teklehaymanot, 2009). The highest informant consensus factor reported for the present study was for tumors by *Cucumis dipsaceus and Jasminum abyssinicum*, which was 0.75, followed by rabies, which was 0.7. The ICF in the current study was relatively low as compared to the previous studies (Mesfin et al., 2009; Enyew et al., 2014). This may be because in the present study, the six districts have been included, which causes the variation in the agreement of medicinal plants used for specific diseases. The knowledge of medicinal plants varies from place to place because the knowledge is localized to a specific area.

The major threats to medicinal plants in the study area were agricultural expansion followed by animal grazing. The present study was in line with the study conducted in Horro Guduru that revealed agricultural expansion was the most common medicinal plants threats (Birhanu et al., 2015). The other studies conducted in different parts of Ethiopia also mentioned agricultural expansion as the main threat to medicinal plants (Mesfin et al., 2009; Bekele and Reddy, 2015; Wubetu et al., 2017). This is due to the rapid population growth in the country and decreasing productivity of the existing land for production. The loss of biodiversity diminishes the supply of raw materials for drug discovery and affects the spread of disease as a source of primary healthcare for 80 percent of the world’s population (Alves and Rosa, 2007). Threats to traditional medicinal plant also cause disappearance of indigenous knowledge about the plants (Calixto, 2005).

In general, the present study helps to preserve the traditional knowledge of medicinal plants in the West Shewa Community. For the scientific community, it could be the basis for picking plant, which could be a candidate for drug discovery and development since many modern drugs originated from traditional medicines. It could also have contributed to the preservation of a culture by documenting the value of the medicinal plants in the studied communities. This study also provides economic opportunity for the communities of West Shewa and the healers those depends on the plants for their livelihood, and the community at large since they use them at an affordable cost in their area of residence.

## Conclusion

The study area was rich in medicinal plants. A total of 51 families of medicinal plants with 108 species were identified. Fabaceae were the most common family used in the West Shewa zone. The traditional healers reported that leaves were commonly used as plant parts for the preparation of the medicine, and most of the traditional medicines prepared were given through oral route of administration. The traditional healers commonly used fresh plants for the preparation of medicine, and squeezing was the most common method of preparation. The major threat to the medicinal plant in the study area was agricultural expansion. Since this is the preliminary study, another study needs to be conducted on the efficacy and safety of the claimed medicinal plant. Additionally, standardization and determination of the dose of medicinal plant needs to be conducted.

## Data Availability

The original contributions presented in the study are included in the article/Supplementary Material, further inquiries can be directed to the corresponding author.
