# Supplementary material for: Ethnobotanical study of traditional medicinal plants used to treat human ailments in West Shewa community, Oromia, Ethiopia
Source: Front Pharmacol. 2024 Jul 19;15:1369480. doi: 10.3389/fphar.2024.1369480 (PMC11294152; doi:10.3389/fphar.2024.1369480)
Supplement: Supplementary file 1 [file Table1.docx]

| **Table S1**: Medicinal plants used for human ailments in selected districts of Southwest Shoa zone Oromia, Ethiopia | | | | | | | | |
| --- | --- | --- | --- | --- | --- | --- | --- | --- |
| Family Name | *Scientific name* | Common Name in Afaan oromo | Disease to be treated | Plant parts used | How to use(dry/ fresh) | Ways of preparation | Root of administration | |
| Acanthaceae | *Justicia schimperiana*  T.Anderson | Dhummuugaa | Emergency | Leaf | Fresh | Maceration | Oral | |
|  |  |  | Febrile illness | Leaf | Fresh | Its leaves together with urgeessaa and hidda reeffaa leaves Boiling with water | Inhalational | |
|  |  |  | Emergency | Leaf | Fresh | Chewing | Oral | |
|  |  |  | Emergency | Leaf | Fresh | Squeezing | Oral | |
| Alliaceae | *Allium sativum* L. | Qullubbi adii | Common cold | Bulb | Fresh | Crushed and mixed with honey | Oral | |
| Amaranthaceae | *Cynoglossum lanceolatum* Forssk. | Maxxannee/ Qaxaxama | postpartum hemorrhage | Root | Fresh | Crushed and mixed with honey or sugar | Oral | |
|  | *Achyranthes aspera* L. | Dabaaqula | To stop bleeding | Leaf | Fresh | Squeezing the leaves | Topical | |
| Anacardiadceae | *Lannea sp.* | Qasamme | Tonsillitis | Leaf | Fresh | Chewing | Oral | |
| Apiaceae | *Anethum graveolens* L. | Insilaalee | Indigestion | Whole plant | Fresh | Decoction | Oral | |
|  |  |  | Urinary retention | Whole plant | Fresh | Decoction | Oral | |
| Apocynaceae | *Landolphia buchananii*  Stapf | Geeboo | Dislocation | Fruit | Fresh | Decoction | Oral | |
|  | *Marsdenia schimperi* Decne. | Gorrisa | Rabies | Root | Fresh | Crushed and mixing with milk | Oral | |
|  | *Carissa spinarum* L. | Hagamsa/Agamsa | Diarrhoea | Leaf | Fresh | Chewing | Oral | |
|  | *Periploca linearifolia* Quart.-Dill. & A.Rich | Aannanoo | Leishmaniasis | Stem | Fresh | Exudate | Topical | |
|  |  |  | Wart | Leaf | Fresh | Exudate | Topical | |
|  |  |  | Leishmaniasis | Stem | Fresh | Exudate | Oral | |
|  |  |  | Leishmaniasis | Stem | Fresh | Exudate | Topical | |
|  |  |  | Wart | Stem/leaves | Fresh | Squeezing | Topical | |
|  |  |  | Wart | Stem/leaves | Fresh | Squeezing | Topical | |
|  |  |  | Wart | Stem/leaf | Fresh | Exudate | Topical | |
|  | *Carissa spinarum* L. | Agamsa | Snake bite | Leaf | Fresh | Squeezing | Oral | |
| Asparagaceae | *Agave sisalana* Perrine | Halgee Lummuxxii | Ear pain | Stem | Fresh | Squeezing to the ear | Topical (directly to the ear) | |
|  | *Asparagus africanus*  Lam. | Saritii | Spider poisoning | Root | Fresh | Crushed and tied on the affected area | Topical |  |
| Asteraceae | *Vernonia auriculifera*  Hiern | Reetii | Burn | Bark | Fresh | By powdering the plant and mixing with Rabbit skin | Topical | |
|  | *Echinops kebericho* Mesfin | Qorobichoo | Stomach ache | Root | Dry | Powder | Oral | |
|  |  |  | Delivered mother | Root | Dry | Powder | Fumigation | |
|  | *Parthenium hysterophorus* L. | - | Tonsillitis | Leaf | Fresh | Squeezed | Oral | |
|  | *Guizotia abyssinica* Cass. | Nuugii | Cough | Seed |  | Decoction of seed | Oral | |
|  |  |  | Asthma | Seed |  | Decoction of seed | Oral | |
|  |  |  | constipation | Seed |  | Decoction of seed | Oral | |
|  | *Acmella caulirhiza*  Delile | Gororsaa/  Bargudii | Tonsillitis | Leaf | Fresh | Squeezing | Eating | |
|  |  |  | Rabies | Root | Fresh | The root crushed and mixed with food /milk | Oral | |
|  |  |  | Rabies | Root | Fresh | Taking by food or milk | Oral | |
|  |  |  | Tonsillitis | Seeds | Fresh | Powder mixed with water | Oral (drunk) | |
|  |  |  |  | seeds/fruit) | Fresh | Powder mixed with water | Oral | |
|  |  |  |  | Seed | Fresh | Decoction | Oral | |
|  | *Vernonia amygdalina*  Del. | Dheebicha/Girawa | Anthrax | Leaf | Dry | Mixed with water | Oral | |
|  |  |  | Anthrax | Leaf | Fresh | dissolving in water | Oral | |
|  |  |  | nasal Bleeding/epistaxis) | Seed | Dry | Smoking | Nasal | |
| Bignoniaceae | *Stereospermum kunthianum* Cham. | Botoroo | Tooth ache | Bark | Fresh | Hold with teeth | Oral | |
| Boraginaceae | *Cynoglossum sp.* | - | Febrile illness | Leaf | Fresh | Fresh leaves are chewed | Oral | |
| Brassicaceae | *Brassica carinata*  A.Braun | Gomanzara/raafuu | Abdominal distension | Seed | Dry | Roosted and powdered mixed with water and drunk | Oral | |
|  | *Lepidium sativum* L. | Feexoo | Helmentiasis | Seed | Dry | Powder mixed with water | Oral | |
|  |  |  | Febrile illness | Seed | Dry | Powder | Oral | |
|  | *Brassica nigra*  W.D.J.Koch | Sanaafica | Abdominal distention | Seed | Dry |  | Oral | |
| Commelinaceae | *Commelina sp.* | Fangasii | Fungi infection in groin area | Leaf/Stem | Fresh | Squeezing the secreation from leaf and stem | Topical | |
| Crassulaceae | *Kalanchoe laciniata*  (L.) DC. | Bosoqqee | Edema | Leaf | Fresh | Fresh leaf heated and applied on the affected area | Topical | |
|  |  |  | Eye disease | Stem | Fresh | Rubbing to the affected area | Topical | |
| Cucurbitaceae | *Peponium vogelii* Engl. | Buqqee seexanaa | Evil sprit | Leaf | Fresh | Inhalation | Inhalation | |
|  | *Cucurbita pepo* L. | Dabaaqula | For treatment of tape worm | Fruit and seed | Fresh | The seed rosted and cooked with fruit and eaten | Oral | |
|  | *Cucumis ficifolius*  A.Rich. | Hidda hooloo | Emergency | Leaf | Fresh | Crushed | Topical | |
|  |  |  | Abdominal cramp | Root | Fresh | Crushed and with food | Oral | |
|  |  |  | Edema | Leaf | Fresh | By dissolving in water | Topical | |
|  | *Cucumis dipsaceus*  Ehrenb. ex Spach | Hidda lafa keessaa | Tumour | root | Fresh | oral/topical | Topical | |
|  |  |  | Anthrax | Leaf | Dry | Powder | Oral | |
|  |  |  | Leishmaniasis | Leaf | Dry | Powder | Topical | |
|  |  |  | Scabies | Leaf | Fresh | Squeezing | Topical | |
|  |  |  | Tumour | Roots | Fresh | If External heating the root and apply to the site of the tumor and if internal chewing | Chewable and Topical | |
|  | *Zehneria scabra* Sond. | Hidda Reeffaa | Leishmaniasis , Skin disorder | Leaf | Dry | Grinding leaf together with Ittachaa leaves | Nasal application or oral | |
|  |  |  | Febrile illness | Leaf | Fresh | Boiling | Inhalational | |
| Ebenaceae | *Euclea racemosa* L. | Mi'eessa | Eye | Leaves | Fresh | Juice | Topical | |
| Euphorbiacea | *Euphorbia ampliphylla* Pax | Adaammii | Haemorrhoids | Stem | Fresh | Exudate | Topical | |
|  |  |  | Haemorrhoids | Leaf | Fresh | Exudate | Topical | |
|  |  |  | Leishmaniasis | Leaf | Fresh | Exudate | Topical | |
|  | *Croton macrostachyus*  Hochst. ex Delile | Bakkanniisa | Tinea Versicolor | Leaf | Fresh | Squeezing | Topical | |
|  |  |  | Tinea Versicolor | Stem | Fresh | Exudate | Topical | |
|  |  |  | Wound infection | Stem | Fresh | Exudate | Topical | |
|  |  |  | Wound infection | Leaves | Fresh | Exudate | Topical | |
|  |  |  | Ring worm | Stem | Fresh | Exudate | Topical | |
|  |  |  | Eczema | Stem | Fresh | Exudate | Topical | |
|  |  |  | To stop bleeding | Leaf | Fresh | Exudate | Topical | |
|  | *Ricinus Communis* L. | Qobboo | Itching | Seeds | Fresh | Mixing with butter | Topical | |
|  |  |  | Leprosy | Seeds | Dry | Powder | Topical | |
|  |  |  | Herpes zoster | Seed | Dry | Powder | Topical | |
|  |  |  | Baldness | Seed | Dry/fresh | By dissolving with better | Topical | |
|  | *Euphorbia schimperiana* Scheele | Allooyee | Haemorrhoids | Latex | fresh | Applied on the affected area | Topical | |
| Fabaceae | *Pterolobium stellatum*  (Forssk.) Brenan | Harangama gurraacha | Toothache | Leaf | Fresh | Leaf as it is | Putting on the tooth | |
|  |  |  | Toothache | Leaf | Dry/fresh | Holding on tooth | applying on tooth | |
|  | *Millettia ferruginea* (Hochst.) Hochst. ex Baker | Birbirraa | Lung disease | Root | Dry | Extracting with water | Oral (drunk) | |
|  |  |  | Asthma/TB | Root | Dry | Powder mixed with water and drunk | Oral | |
|  | *Calpurnia aurea*  (Aiton) Benth. | Manyaar | Toothache | Leaf | Fresh | Putting on the tooth | Oral | |
|  |  |  | Toothache | Leaf | Fresh | Chewing | Oral | |
|  | *Desmodium sp.* | Hidda hantuutaa | Infected Wound | Leaf | Fresh | Heating with fire | Topical | |
|  | *Indigofera spicata*  Forssk. | Qoricha sinbira halkanii | Leishmaniasis  Leishmaniasis | Leaf | Dry | Powder | Inhalation | |
|  |  |  |  | Leaf | Dry | Powder | Nasal | |
|  | *Trifolium pichisermollii* J.B.Gillett | Siddisa | Spider poisoning | Whole plant | Fresh | The whole plant powderd and applied with butter | Topical | |
|  | *Acacia albida* Rojas Acosta | Garbii | To treat eye disease | Leaf | Fresh | Squeezed the affected area | Topical | |
|  | *Vicia faba* L. | Baaqelaa | Ascariasis | Seed | Dry | Decoction | Oral | |
| Icacinaceae | *Apodytes dimidiata* E.Mey. ex Arn. | Calalaqaa | Cough | Leaf | Fresh | Decoction | Oral | |
| Labiatae | *Premna schimperi* Engl | Urgessa | Toothache | Leaf | Fresh | Squeezing | Buccal | |
|  |  |  | Toothache | Leaf | Fresh | Chewing | Buccal | |
|  |  |  | Toothache | Leaf | Fresh | Holding on tooth | Buccal | |
|  |  |  | Febrile illness | Leaf | Fresh | Boiling | Inhalation | |
|  |  |  | Febrile illness | Leaf | Fresh | Squeezing | Topical | |
| Lamiaceae | *Ajuga integrifolia*  Buch.-Ham. | Harma guusaa | Stomachache | Leaf | Fresh | Squeezed | Oral (drunk) | |
|  |  |  | Abdominal cramp, deworming | Leaf | Fresh | Squeezed | Oral | |
|  |  |  | Abdominal cramp | Leaf | Fresh | Mixed with water | Oral | |
|  |  |  | Abdominal cramp | Leaf | Fresh | Maceration | Oral | |
|  | *Thymus schimperi*  Ronniger | Xoosinyii | Hypertension | Leaf | Fresh | Infusion | Oral | |
|  | *Leonotis nepetifolia*  (L.) R.Br. | Bokkolluu | Fever | Leaf | Fresh | Squeezing | Oral | |
|  | *Ocimum basilicum* L. | hancabbi/damakasee | Febrile illness | Leaf | Fresh | Squeezing/juice | Topical | |
|  |  |  | To stop bleeding | Leaf | Fresh | Squeezing the leaves | Topical | |
|  |  |  | Febrile illness | Leaf | Fresh | Squeezing | Inhalation | |
|  |  |  | Herpes simplex | Leaf | Fresh | Squeezing | Topical | |
|  |  |  | Febrile illness | Leaf | Fresh | Squeezing | Oral | |
|  |  |  | Febrile illness | Leaf | Fresh | Squeezing | Nasal | |
|  |  |  | For delivered mother | Leaf | Fresh | Maceration | Nasal | |
|  |  |  | Wound healing | Leaf | Fresh | Squeezing | Topical | |
|  | *Rosmarinus officinalis* L. | Baala xibsii | Treatment of burn infection | Leaf | Fresh | Squeezed to the affected area | Topical | |
|  | *Rydingia integrifolia* (Benth.) Scheen & V.A.Albert | Xunjiitii | Febrile illness | Leaf | Fresh /dry | Fumigation | Inhalation | |
| Linaceae | *Linum usitatissimum* L. | Talba | Constipation | Seed | Dry | Decoction | Oral | |
| Loganiaceae | *Nuxia congesta*  R.Br. ex Fresen. | Qaqawwee | Toothache | Bark |  | Chewing and holding in the mouth | Oral | |
|  | *Buddleja polystachya* | Anfara | Ascariasis | Leaf | Fresh | Maceration | Oral | |
| Malvaceae | *Pavonia urens* Cav. | Hincinni | Tonsillitis | Leaf | Fresh | Infusion | Oral | |
|  | *Cyathula uncinulata* (Schrad.) Schinz | Maxxannee | To stop bleeding | Leaves | Fresh | Squeezing the leaves | Topical | |
|  | *Pavonia urens*  Cav. | Sam'ee | Febrile illness | Leaf | Fresh | Squeezing | Topical | |
|  | *Malva verticillata* L. | Littii | Tape worm | Root | Fresh | The root is washed then crushed and drunk | Oral | |
| Meliaceae | *Melia azedarach* L. | Mimi | Toothache | Leaf | Fresh | Putting on the affected teeth | Buccal | |
|  | *Azadirachta indica*  A.Juss. | Nim tree (EN) | Tape worm | Leaf | Fresh | The leaf crushed and mixed with water then filtered | Oral | |
| Melianthaceae | *Bersama abyssinica* Fresen. | Araarsaa | Abdominal cramp | Root | Fresh | The fresh root crushed and mixed with food | Oral | |
| Mensipermaceae | *Stephania abyssinica*  Walp. | Kalaalaa | Anthrax | Root | Dry | Crushed | Topical | |
|  |  |  | Anthrax | Root | Dry | Dissolving in water and staying for long time | Oral | |
| Moraceae | *Ficus carica* L. | Lugoo | Hemorrhoids | Fruit | Fresh | Crushed and applied on the affected area | Topical | |
|  | *Ficus sur*  Forssk. | Harbuu | Impotency | Root | Fresh | Decoction | Oral | |
|  |  |  | Kidney infection | Bark | Fresh | Maceration | Oral | |
| Myrisinacea | *Embelia schimperi*  Vatke | Hanquu | Tinea Infection (fungal infection ) | Seed | Dry | Decoction | Oral | |
|  |  |  | Leprosy | Root | Fresh | Crushed and mixed with water | Oral | |
| Myrtaceae | *Eucalyptus globulus*  Labill. | Baargamoo Adii | Diarrhoea | Leaf | Fresh | Squeezing | Oral | |
|  |  |  | Common cold | Leaf | Fresh | Decoction(the leaf is boiled and inhaled | Inhalation | |
|  | *Eucalyptus camaldulensis*  Dehnh. | Bargamo diimaa | Febrile illness | Leaf | Fresh | Fumigation | Inhalation | |
| Oleacea | *Jasminum abyssinicum*  Hochst. ex DC. | Misirichii | Anthrax | Leaves and root | Fresh | Squeezing | Oral | |
|  |  |  | Tumour | Leaves | Fresh | Squeezing | Topical | |
|  | *Olea europaea* L. | Ejersa | Snake bite | Leaf | Fresh | Maceration | Oral | |
| Oliniaceae | *Olinia rochetiana* A.Juss. | Solee | Scabies | Leaf | Fresh | Squeezed | Oral | |
| Phytolaccaceae | *Phytolacca dodecandra*  L'Hér. | Handoodee | Rabies | Root | Dry/fresh | With milk or honey | Oral | |
|  |  |  |  |  | Dry | Powder | Oral | |
|  |  |  |  |  | Dry | Powder mixed with milk or meat | Oral | |
|  |  |  |  |  | Fresh | Powder mixed With milk or meat | Oral | |
|  |  |  |  |  | Fresh | With milk | Oral | |
|  |  |  |  |  | Fresh | By eating with food | Oral | |
|  |  |  |  |  | Fresh | Mixed with food | Oral | |
| Plantaginaceae | *Plantago lanceolata* L. | Qorxobbii/Qorrisa | Wound | Leaf | Fresh | Squeezing | Topical | |
|  |  |  | To stop bleeding | Leaf | Fresh | Extraction by pressure | Topical | |
|  | *Calpurina aurera (Lam.) Benth* | Cheekaa | Abdominal cramp | Leaf | Fresh | Dissolved in milk | Oral | |
|  |  |  | Diarrhoea | Seed | Dry | Powder | Oral | |
| Poaceae | *Chloris gayana*  Kunth | Cingiitii/Coqorsa | Respiratory problem | Leaf | Dry/fresh | Powder | Inhalation | |
|  |  |  | Measles | Leaf | Fresh | The leaf chewed spit | Oral | |
|  | *Cymbopogon citratus*  (DC.) Stapf | Xajjisaara | Evil sprit | Leaf | Fresh | Eaten (alanfatama) | Oral | |
|  | *Hordeum vulgare* L. | Garbuu | Broken bone | Seed | Dry | Powdered and eaten as powrage | Oral | |
| Polygonaceae | *Rumex nepalensis* Spreng. | Tultii/Tibijjii | Measles | Leaf | Fresh | Maceration | Topical | |
|  |  |  | Desert wound | Root | Dry | Powder | Topical | |
|  |  |  | abdominal cramp | Root | Fresh | Maceration | Oral /drunk | |
|  |  |  | Abdominal cramp, Deworming | Root | Fresh | Crushed and mixed with food | Oral | |
|  |  |  | abdominal cramp | Root | Fresh | Crushed and taken raw | Oral | |
|  |  |  | Desert wound | Root | Fresh | Maceration | Topical | |
|  |  |  | abdominal cramp | Root | Fresh | Chewed | Oral | |
|  | *Rumex nervosus* Vahl. | Dhangaggoo | haemorrhoids | Leaf | Dry | Maceration | Topical | |
| Ranunculaceae | *Clematis longicauda*  Steud. ex A.Rich. | Hidda adii | Anthrax | Leaf | Dry | Dissolving in water and staying for long time | Oral | |
|  | *Clematis simensis* Fresen. | Hidda Feetii | Liver disease | Leaf | Dry | Powder | Oral | |
|  |  |  | Skin disorder | Leaf | Dry | Powder | Topical | |
|  |  |  | Febrile | Leaves | Fresh | Squeezing | Oral | |
|  |  |  | Toothache | Leaf | Dry/fresh | By dissolving in coffee | Oral | |
|  |  |  | Haemorrhage | Root | Dry | Crushed | Topical | |
|  | *Nigella sativa* L. | Abasuuda | Tonsillitis | Seed | Dry | Powder | Oral | |
|  |  |  | Ameobiasis | Seed | Dry | Decoction | Oral | |
| Rhamnaceae | *Rhamnus prinoides*  L'Hér. | Geeshoo | Tonsillitis | Leaf | Fresh | Chewing | Oral | |
|  |  |  | Gonorrhoea | Leaf | Fresh | Powder dissolved in water | Oral (drunk), bathing | |
|  |  |  |  |  | Dry | Powder | Oral | |
| Rosaceae | *Prunus Africana*  (Hook.f.) Kalkman | Hoomii | Wound healing | Bark | Dry | Decoction | Oral | |
|  |  |  | Urinary retention | Bark | Dry | Decoction | Oral | |
| Rubiaceae | *Coffea arabica*L. | Buna | Diarrhoea | Seed | Dry | Decoction | Oral | |
|  | *Rubus steudneri*  Schweinf. | Goraa | Leishmaniasis, Skin disorder | Leaf | Dry | Crushed with leaves of Ittacha and hidda reeffaa | Oral | |
|  |  |  | Gonorrhoea | Leaf | Fresh | Squeezing | Bathing | |
|  | *Gardenia ternifolia*  Schumach. & Thonn. | Gambeela | Evil eye | leaves | Fresh | Maceration | Oral | |
|  | *Rubus steudneri*  Schweinf. | Goraa | Leishmaniasis, Skin disorder | Leaf | Dry | Crushed with leaves of Ittacha and hidda reeffaa | Oral | |
|  |  |  | Gonorrhoea | Leaf | Fresh | Squeezing | Bathing | |
| Rutaceae | *Citrus limon*  (L.) Osbeck | Loomii | Common cold | Fruit | Fresh | Squeezed and drunk | Oral | |
|  | *Clausena anisata*  (Willd.) Hook.f. | Ulmaa | Anthrax | Leaf | Dry | mixed with water | Oral | |
|  |  |  | Anthrax | Leaf | Dry | Maceration dissolving in water and staying for long time | Oral | |
|  | *Ruta chalepensis* L. | Cillaaddama | Abdominal cramp | Leaf | Fresh | Infusion | Oral | |
|  |  |  | Stomachache | Leaf | Fresh | Eaten (alanfatama) | Oral | |
|  | *Teclea nobilis*  (Delile) Mziray | Hadheessa | Diabetes mellitus | Leaf | Fresh | Infusion | Oral | |
| Salicaceae | *Salix mucronata* Thunb. | Alaltuu | Anthrax | Leaf | Dry | Mixed with water | Oral | |
|  |  |  |  |  |  | Dissolving in water and staying for long time | Oral | |
| Sapindaceae | *Dodonaea angustifolia* L.f | Itacha/ Kitkittaa | Respiratory problem | Leaf | Dry/fresh | Fumigation | Inhalation | |
|  |  |  | Diarrhoea | Leaf | Fresh | Squeezing | Oral | |
|  |  |  |  |  |  |  |  | |
| Scrophulariaceae | *Verbascum sinaiticum*  *Benth.* | Gurra harree/qaxaxannaa | Rabies | Root | Fresh | With food | Oral | |
|  |  |  | Anthrax | Root | Dry | Mixed with water | Oral | |
|  |  |  | Abdominal cramp | Root | Fresh | Chewing | Oral | |
|  |  |  | Anthrax | Root | Dry | Dissolving in water and staying for long time | Oral | |
| Simaroubaceae | *Brucea antidysenterica*  J.F.Mill. | Qomonyo | Rabies | Seed | Fresh | Crushed | Oral | |
|  |  |  | Rabies | Seed | Fresh | Mixed with food | Oral | |
| Solanaceae | *Datura stramonium* L. | Asangira/Manjii | Tonsillitis | Leaf | Fresh | Squeezing | Oral | |
|  |  |  | Allergic reaction | Leaf | Fresh | Squeezing | Topical | |
|  |  |  | Allergic reaction | Leaf | Fresh | Maceration | Topical | |
|  |  |  | Dyspepsia | Leaf | Fresh | Squeezing | Oral | |
|  | *Withania somnifera* (L.) Dunal | Gizawwaa | Abdominal cramp | Root | Fresh | Crushed and mixed with food | Oral | |
|  |  |  | Deworming | Root | Fresh | Crushed and mixed with food | Oral | |
|  | *Solanum anguivi* Lam | Hiddi adii | Anthrax | Leaf | Dry | Mixed with water | Oral | |
|  | *Solanum campylacanthum* Hochst. ex A.Rich. | Hiddii/horgocca | Tonsillitis | Fruit | Fresh | Squeezing | Oral | |
|  |  |  | Tonsillitis | Fruit | Fresh | Squeezing | Oral(poured to the esophagus of the child | |
|  | *Capsicum annuum* L. | Qariya(AM) | Common cold | Fruit | Fresh | Copped and mixed with onion and garlic and eaten | Oral | |
|  | *Nicotiana tabacum* L. | Tamboo | Hepatitis | Leaf | Dry | Decoction | Inhalation |  |
| Urticaceae | *Girardinia bullosa*  (Hochst. ex Steud.) Wedd. | Doobbii | Syphilis | Root | Fresh | Powder mixed with water | Bathing | |
|  |  |  | Gonorrhoea | Root | Fresh | Squeezing | Bathing | |
|  |  |  | Syphilis | Root | Fresh | Maceration | Bathing | |
|  |  |  | Syphilis | Root | Fresh | Maceration | Bathing | |
|  | *Urera hypselodendron*  (Hochst. ex A.Rich.) T.Wells & A.K.Monro | Dhoqonuu | Urinary retention | Leaf | Fresh | Decoction | Oral | |
| Verbenaceae | *Verbena officinalis* L. | Atoch/  Baala waraantii | Febrile illness | Leaf | Fresh | Squeezed and drunk | Oral | |
|  |  |  | Abdominal cramp | Leaf | Fresh | Squeezed and drunk | Oral | |
|  |  |  | Dyspepsia | Stem | Dry | Powder | Oral | |
|  |  |  | Dyspepsia | Dry | Stem | Powder | Oral | |
| Vitaceae | *Cyphostemma sp.* | Amichoo | Abdominal cramp | Root | Fresh | Squeezing | Oral, topical | |
|  |  |  | Rabies | Root | Dry | Powder | given orally with coffee | |
| Zingibilaceae | *Zingiber officinale* Roscoe | Zinjibila | Tonsillitis and stomachache | Stem | Fresh | Powder | Oral | |
|  |  |  | Abdominal pain | Roots | Fresh | Chewing | Oral | |
