# Supplementary material for: Ethnobotanical study of traditional medicinal plants used to treat human ailments in West Shewa community, Oromia, Ethiopia
Source: Front Pharmacol. 2024 Jul 19;15:1369480. doi: 10.3389/fphar.2024.1369480 (PMC11294152; doi:10.3389/fphar.2024.1369480)
Supplement: Supplementary file 2 [file DataSheet1.docx]

**Annexes**:

1. **Questionnaires**

**Part I: Socio-demographic characteristics of the Respondents**

**Instruction:** Please encircle the respondents’ responses from the given options and write their response with legible hand writing.

Name of data collector---**----------------------------------------------------------------------------**

| S.no | A. Background information | |
| --- | --- | --- |
|  | Zone: | 1. South West Shewa 2. Special zone surrounding Finfinne |
|  | Districts | ____________________________ |
|  | Address | 1. Rural 2. Urban |
|  | Sex | 1. Male 2. Female |
|  | Age in years | ____________years |
|  | Religion of the respondents | 1. Orthodox 2. Protestant 3. Muslim 4. Others specify______________________ |
|  | Ethnicity | 1. Oromo 2. Amhara 3. Gurage 4. Others (specify)------ |
|  | Education background | 1. Has no formal education 2. Primary education 3. Secondary school 4. College/diploma 5. University/ first degree or above |
|  | Marital status | 1. Married 2. Unmarried 3. Divorced 4. Widowed |
|  | Occupation of the respondents | 1. Farmer 2. Private employee 3. Government employee 4. Others, specify ________________________ |
|  | Average monthly house-hold income in birr | _______________________________ |
|  | Monthly income from medicinal plants as a traditional healer? (ETB) |  |
|  | Years of experience as a traditional healer | _______________________ years |
|  | From whom you initially acquired this, knowledge? | 1. Family members: Specify: ______________ 2. Friends 3. Religious persons 4. God gifts 5. Others specify: ____________________ |
|  | Have you had any training on traditional medicine? | 1. Yes 2. No |
|  | If yes on what topics you had training? Specify the areas training was given. | ______________________ |
|  | Who provided you the training? | ______________________ |
|  | Mode of practice on this traditional medicine. | 1. Full time 2. Part time |
|  | Number of patients you provided medicinal plants per week? | ---------------------------------------------- |

1. **List the medicinal plants you used for the treatments of human ailments?**

| **S. No.** | **Medicinal plants used** | **Remark** |
| --- | --- | --- |
|  |  |  |
|  |  |  |
|  |  |  |
|  |  |  |
|  |  |  |
|  |  |  |
|  |  |  |
|  |  |  |
|  |  |  |
|  |  |  |
|  |  |  |
|  |  |  |
|  |  |  |

1. **Could you tell me the use of medicinal plants you mentioned above? (Use new row if more than one plant parts is used by traditional healers) Please use the back of the paper if more.**

|  | **Local name** | **Name of the disease treated by the plants.** | **Plant’s part used (Leaf, Stem, root, bark, flower, seeds, fruits and others specify)** | **How to use? Fresh or dried)** | **Ways of preparation? (Powder, juices, with coffee/tea, maceration, with food, and others specify)** | **Days of collection in a week? (all the day, name of specific day)** | **Time of collection? (Morning, midday, afternoon night)** | | **Growth Habit (**tree, shrub, herb, and climber) | | **Route of administration? (Topical, oral, parenteral, buccal, inhalation, nasal, and others specify)** | **Dosage measurement. (By spoon, finger, cup, palm and others specify)** | **Storage conditions (specify)** |
| --- | --- | --- | --- | --- | --- | --- | --- | --- | --- | --- | --- | --- | --- |
|  |  |  |  |  |  |  | |  | |  |  |  |  |
|  |  |  |  |  |  |  | |  | |  |  |  |  |
|  |  |  |  |  |  |  | |  | |  |  |  |  |
|  |  |  |  |  |  |  | |  | |  |  |  |  |
|  |  |  |  |  |  |  | |  | |  |  |  |  |
|  |  |  |  |  |  |  | |  | |  |  |  |  |
|  |  |  |  |  |  |  | |  | |  |  |  |  |
|  |  |  |  |  |  |  | |  | |  |  |  |  |
|  |  |  |  |  |  |  | |  | |  |  |  |  |
|  |  |  |  |  |  |  | |  | |  |  |  |  |
|  |  |  |  |  |  |  | |  | |  |  |  |  |
|  |  |  |  |  |  |  | |  | |  |  |  |  |
|  |  |  |  |  |  |  | |  | |  |  |  |  |
|  |  |  |  |  |  |  | |  | |  |  |  |  |
|  |  |  |  |  |  |  | |  | |  |  |  |  |
|  |  |  |  |  |  |  | |  | |  |  |  |  |
|  |  |  |  |  |  |  | |  | |  |  |  |  |
|  |  |  |  |  |  |  | |  | |  |  |  |  |

|  | On traditional medicine from the plant is there any additional ingredient added apart from medicinal plant | 1. Yes 2. No |
| --- | --- | --- |
|  | If yes what is its function? | 1. Excipient 2. Medicinal value |
|  | What are you going to do if treatment fails upon the use of the drug? |  |
|  | Containers used for storage of the formulations? |  |
|  | For how long can you keep the formulation without deterioration? |  |
|  | Is there any observed Traditional medicine interaction? (If no skip to question # 26) | 1. Yes 2. No |
|  | If yes with what it has interaction? | 1. With other traditional medicine (specify) 2. With modern medicine (specify) 3. With food (specify) 4. With drinks (specify) 5. Others (specify) |
|  | Do want to transfer this indigenous knowledge to others? | 1. Yes 2. No |
|  | If yes for whom you planned to transfer this indigenous knowledge? | _____________________________________ |
|  | If no to the question # 27 why? | ____________________________________ |
|  | Are there any medicinal plants which are endangered or disappeared? (Any plants previously in use) | 1. Yes 2. No |
|  | If yes specify the name of those medicinal plants which are currently endangered or disappeared? | ________________________________. |
|  | What are the reasons for the threatened or endangered plants? | 1. Using for food 2. Using for construction 3. For charcoal or fuel 4. For furniture 5. Expansion of land farms 6. Climate change 7. Grazing 8. Others Specify: |
